# Characterization of Ebola Virus Mucosal Challenge Routes in Cynomolgus Macaques

**DOI:** 10.1128/jvi.01888-22

**Published:** 2023-03-28

**Authors:** Dylan M. Johnson, Trevor Brasel, Shane Massey, Jeanon Smith, Tania Garron, Shannon Wallace, Xiaoying Yu, David W. Beasley, Jason E. Comer

**Affiliations:** a Department of Microbiology and Immunology, University of Texas Medical Branch, Galveston, Texas, USA; b Office of Regulated Nonclinical Studies, University of Texas Medical Branch, Galveston, Texas, USA; c Experimental Pathology Laboratories Inc., Sterling, Virginia, USA; d Department of Biostatistics & Data Science, University of Texas Medical Branch, Galveston, Texas, USA; University of Kentucky College of Medicine

**Keywords:** Ebola virus disease models, conjunctival Ebola virus infection, oral Ebolavirus infection, filovirus animal models, animal models, Ebola virus, filovirus

## Abstract

*Zaïre ebolavirus* (EBOV) causes Ebola virus disease (EVD), a devastating viral hemorrhagic fever in humans. Nonhuman primate (NHP) models of EVD traditionally use intramuscular infection with higher case fatality rates and reduced mean time-to-death compared to contact transmission typical of human cases of EVD. A cynomolgus macaque model of oral and conjunctival EBOV was used to further characterize the more clinically relevant contact transmission of EVD. NHPs challenged via the oral route had an overall 50% survival rate. NHPs challenged with a target dose of 1 × 10^2^ PFU or 1 × 10^4^ PFU of EBOV via the conjunctival route had 40% and 100% mortality, respectively. Classic signs of lethal EVD-like disease were observed in all NHPs that succumbed to EBOV infection including viremia, hematological abnormalities, clinical chemistries indicative of hepatic and renal disease, and histopathological findings. Evidence of EBOV viral persistence in the eye was observed in NHPs challenged via the conjunctival route.

**IMPORTANCE** This study is the first to examine the Kikwit strain of EBOV, the most commonly used strain, in the gold-standard macaque model of infection. Additionally, this is the first description of the detection of virus in the vitreous fluid, an immune privileged site that has been proposed as a viral reservoir, following conjunctival challenge. The oral and conjunctival macaque challenge model of EVD described here more faithfully recapitulates the prodrome that has been reported for human EVD. This work paves the way for more advanced studies to model contact transmission of EVD, including early events in mucosal infection and immunity, as well as the establishment of persistent viral infection and the emergence from these reservoirs.

## BACKGROUND

*Zaïre ebolavirus* (EBOV) of the *Filoviridae* family is the causative agent of Ebola virus disease (EVD). EVD is a viral hemorrhagic fever that typically presents with high fever, diffuse intravascular coagulation (DIC) leading to clotting deficiencies which may lead to bleeding including hematemesis and bloody diarrhea, myositis, arthralgia, hepatic and renal disfunction, pulmonary edema, fatigue, headache and encephalitis, and death (1, 2). EVD has an average case fatality rate (CFR) of 44%, with individual outbreak CFRs of up to 90%, however, contributing factors to various CFRs has yet to be elucidated (1). It is estimated that EBOV has emerged about 30 times from zoonotic reservoirs since its discovery in 1976 (1, 3), and typically spreads through direct contact in domestic, clinical, and mortuary settings during outbreaks (4–7). Outbreaks of EVD have occurred regularly in Central Africa since the emergence of EBOV (1).

Detailed molecular analysis of clinical EVD has been difficult considering both the local health care capacity and the difficulties of handling highly infectious patient samples. EBOV is a risk group 4 pathogen which requires maximum containment biosafety level 4 (BSL4) laboratories for safe handling, further complicating EBOV research. The 2013 to 2016 outbreak provided the first chance to gain large scale sequence and host-response data from EVD, although these studies are ongoing and are far from providing a complete picture of EBOV pathogenesis in humans (8, 9).

Nonhuman primates (NHPs), specifically cynomolgus macaques (Macaca fascicularis) or rhesus macaques (M. mulatta), are the gold standard laboratory model of EVD (10–12). Studies using these NHPs are typically limited by both the high cost of animals and limited BSL4 lab space availability. As a result, NHP models typically employ intramuscular (IM) challenge with EBOV doses of 1 × 10^3^ PFU which typically results in a uniformly lethal disease (10, 13). This increases statistical power and has led to many insights about EBOV host-pathogen interaction and the development of medical countermeasures against EVD, including an FDA approved vaccine (14). However, in the NHP model, as few as 0.01 plaque forming unit (PFU) of EBOV given IM has been demonstrated to cause lethal disease (15). In humans, the infectious dose for EVD is poorly characterized, although it appears to be less than 1 × 10^1^ PFU in the case of percutaneous inoculation (16, 17).

However, the typical IM inoculation of 1 × 10^3^ PFU of EBOV is a contrived exposure method because EBOV is typically transmitted through direct contact either with the conjunctiva or mucosal surfaces in most human infections (4, 17, 18). An exception to this can be found in the 1976 outbreak of EVD where reusing of needles resulted in 85 cases with a 100% CFR from patients injected with EBOV contaminated needles (19). Additionally, several cases of laboratory acquired infection have further shown the potential of parenteral inoculation to transmit EBOV in humans (20). Further complicating the situation is the observation that increasing doses of EBOV in NHP models tend to correlate with shorter mean survival time (21). Interestingly, it has been suggested that vaccines which preferentially elicit mucosal immunity may play an important role in the protection from natural routes of EBOV transmission (22). Different host-interactions likely contribute to the modified disease phenotypical characteristics of IM injection and direct contact transmission. In direct contract transmission EBOV probably first infects mucosal macrophages and dendritic cells, coopting host antiviral responses, and exploiting the trafficking of infected dendritic cells as a means to enter systemic circulation (2, 23, 24). NHP models of EVD potentially bypass this process by introducing the virus more quickly into systemic circulation through the disruption of capillary beds during IM injection.

Very few animal studies have examined contact transmission as a route of EBOV infection (11). In a ferret model of EVD, oral inoculation of 1 × 10°, 1 × 10^1^, or 1 × 10^2^ PFU caused uniformly lethal disease with a delayed time to death in the 1 PFU challenge group, while conjunctival inoculation of 1 × 10°, 1 × 10^1^, or 1 × 10^2^ PFU did not cause lethal disease (25). Exposure of rhesus macaques to 1.58x10^5^ PFU of EBOV by either oral or conjunctival routes (with 4 monkeys per route of exposure) resulted in uniformly lethal exposure between 7 and 8 days postexposure (26). Cynomolgus macaques exposed to 1 × 10^1^ PFU of EBOV via oral and conjunctival routes (with 2 monkeys per route of exposure) did not develop disease and only 1 of the orally exposed animals seroconverted; exposure to 1 × 10^2^ PFU (1 monkey per route of exposure) resulted in lethal disease for oral exposure and a febrile illness for conjunctival exposure (27). In another study of conjunctival infection with EBOV, 1 × 10^4^ PFU was lethal in 6/6 cynomolgus macaques, while doses of 1 × 10^2^ and 5×10^2^ PFU were only lethal in 1/6 monkeys (28). Here, we investigate both low (1 × 10^2^ PFU) and high (1 × 10^4^ PFU) doses of EBOV in cynomolgus macaques with both oral and conjunctival challenge. The Kikwit strain of EBOV was used, rather than the Mayinga or Makona strains investigated in previous NHP mucosal models of EVD (26–28), because the Kikwit strain is the best characterized strain across all EVD NHP challenge models (29, 30). Importantly, this study is the first to model mucosal transmission of the Kikwit strain of EBOV and has appropriate sample size (*n* = 5 per group) and infectious dose ranges to model partially lethal viral challenge. This study bridges critical gaps in dosing and viral strain in the modeling of mucosal EVD transmission in NHPs. This is crucial to understanding natural filovirus mucosal transmission which is the primary mode of transmission during most outbreaks.

## RESULTS

**FIG 1:**
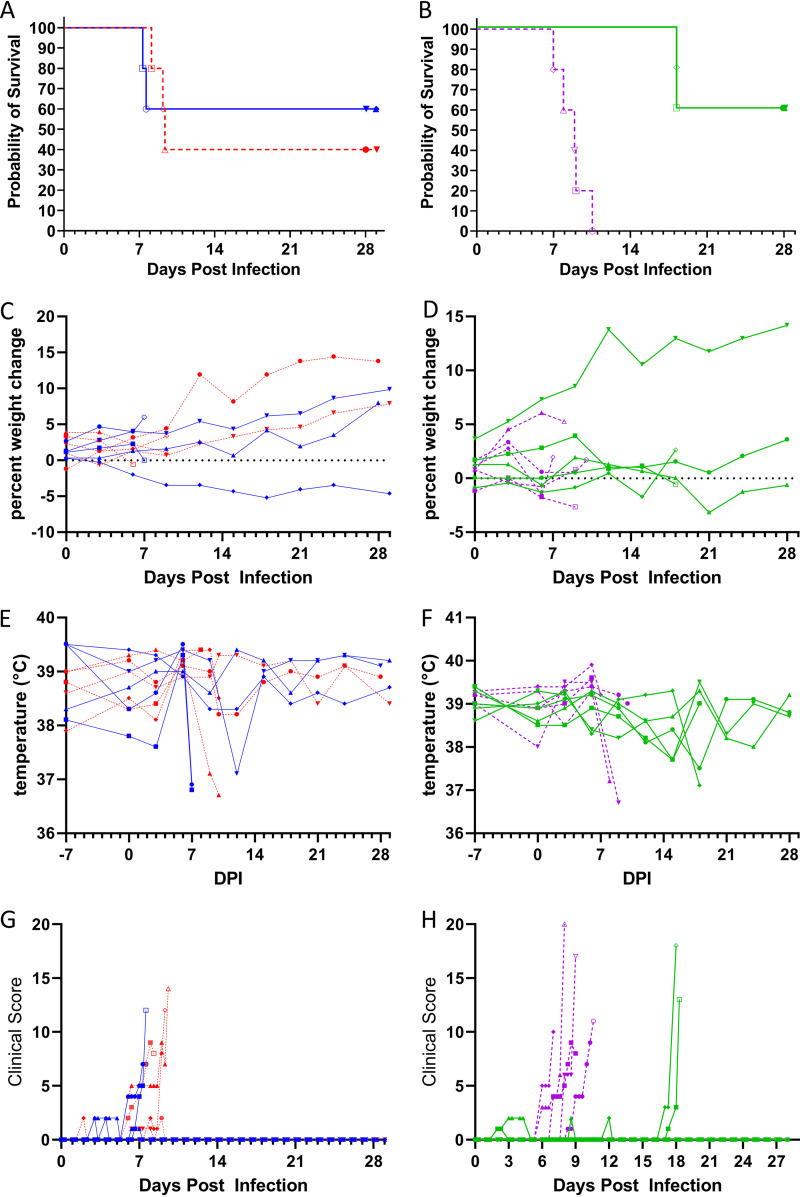
NHPs were challenged with 1 × 10^2^ PFU (blue and green solid connecting lines) or 1 × 10^4^ PFU (red and purple dashed connecting lines) of EBOV at 0 days postinfection (DPI) via the sublingual mucosa (A, C, E, and G) or via the conjunctiva (B, D, F, and H) and monitored for 4 weeks for survival (A and B), percent weight change from baseline measured 7 days prior to challenge (C and D), rectal temperature (E and F), and clinical score (G and H) (*n* = 5). Weights and temperatures were recorded 1 week prior to challenge, at an interval of 3 days following challenge, and prior to euthanasia. Clinical scoring was conducted twice daily from the time of challenge with a third score collected for animals scoring >4 during the second observation. Open symbols represent terminal measurements. Symbols (circles, squares, triangles, diamonds, and inverted triangles) can be attributed to individual animals with the key in Fig. 7. The Kaplan-Meier method was used to estimate differences in the survival function (A and B), the probability that an animal survives over time after challenge with an overall significance of *P = *0.0365 across all groups.

Following challenge, 2/5 NHPs from low dose oral challenge, 3/5 NHPs from the high dose oral challenge, 2/5 NHPs from the low dose conjunctival challenge, and 5/5 NHPs from the high dose conjunctival challenge succumbed. There were not apparent differences in the time to death between the low and high oral dose groups (Fig. 1A), however, there was about a 1-week delay in time to death between the low and high conjunctival doses (Fig. 1B).

There was a slight general trend for weight loss in all groups between days 3 and 6 postchallenge with recovery in survivors by day 9 (Fig. 1C and D). Body temperatures were increased on day 6 in both high and low dose oral challenge groups (Fig. 1E) and the high dose conjunctival dose group (Fig. 1F). Animals meeting endpoint criteria tended to have mild hypothermia. One survivor from each of the low dose oral and low dose conjunctival challenge groups had decreased body temperature on day 12 and 18, respectively. Of the 3 survivors in the low dose conjunctival challenge group, two had elevated temperature and one had low body temperature at day 18 postchallenge.

A few NHPs had clinical scores of 1 or 2 on days 2 to 5 postchallenge related to reduced appetite (Fig. 1G and H). Clinical scores >3 tended to be an early indicator of lethal disease.

**FIG 2:**
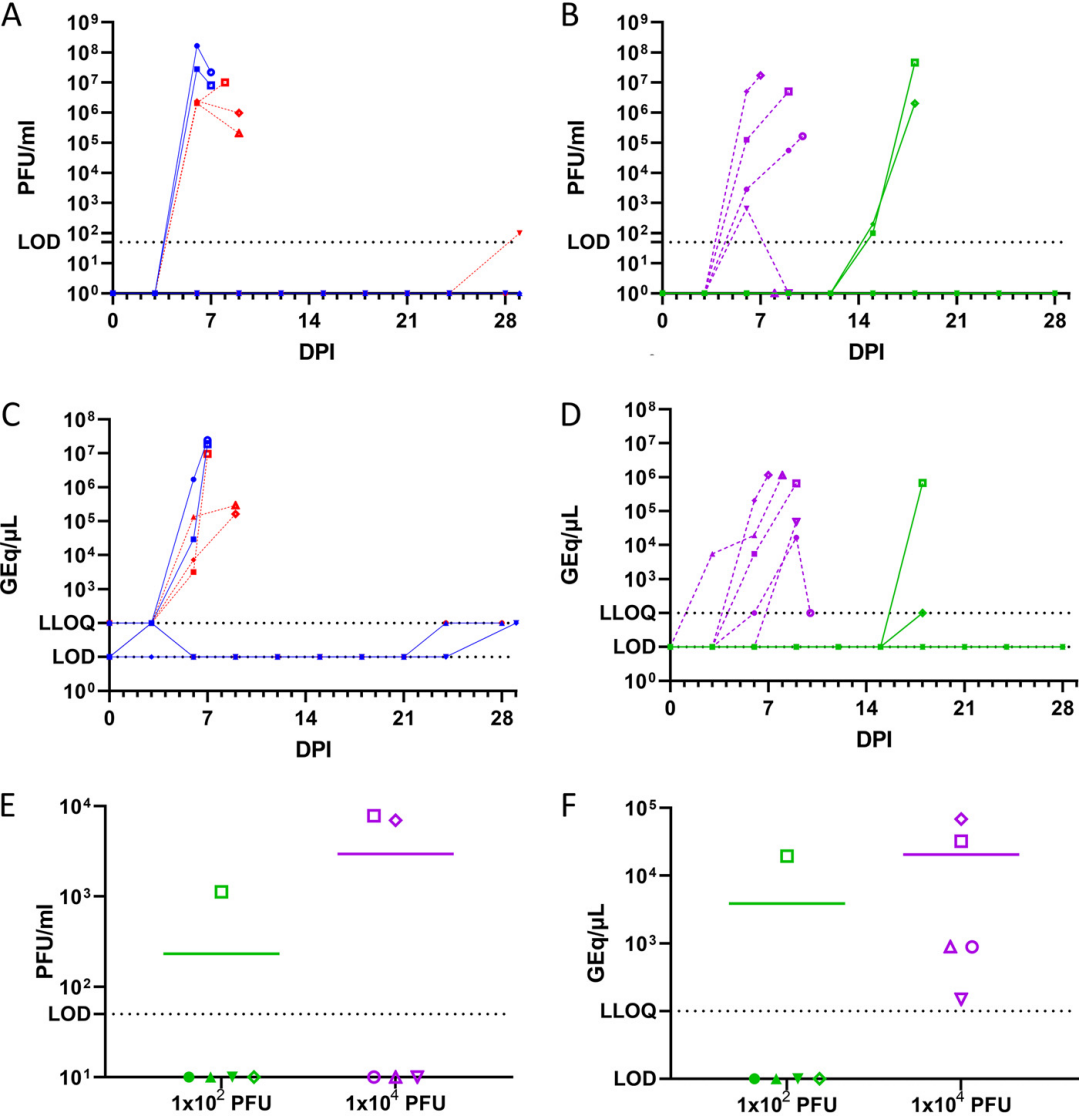
Blood was collected at an interval of 3 days following challenge and prior to euthanasia and used to isolate serum (A, B, C, and D). Vitreous fluid (E and F) was collected during necropsy. Viremia was determined by plaque assay (A, B, and E) or by qRT-PCR (C, D, and F) from NHPs challenge via the oral (A and C) or conjunctival (B, D, E, and F) routes with 1 × 10^2^ PFU (blue and green with solid connecting lines) or 1 × 10^4^ PFU (red and purple with dashed connecting lines) of EBOV at 0 DPI (A, B, C, and D) or following euthanasia (E and F). The limit of detection (LOD) was 50 PFU/mL for plaque assay. Samples below the LOD line for plaque assays did not produce any plaques at the dilutions tested. The lower limit of quantification (LLOQ) for qRT-PCR was 1,000 GEq/μL. Samples with a detected cycle-threshold value within 45 cycles but less than 1,000 GEq/μL are depicted on the LLOQ line. Samples where no amplification was observed in qRT-PCR are depicted on the LOD line. Symbols (circles, squares, triangles, diamonds, and inverted triangles) can be attributed to individual animals with the key in Fig. 7.

NHPs infected with EBOV via the oral route had high viremia detectable by plaque assay (Fig. 2A) and qRT-PCR (Fig. 2C) in all animals that succumbed to infection which was detectable 1 to 3 days prior to meeting endpoint criteria. Viremia tended to remain high in endpoint measurements. A low level of viral RNA was detected in one animal each from the high and low groups at day 24 postchallenge and at the end of the study, with live virus recoverable from the serum of survivor in the high dose at day 29 postchallenge.

A similar trend in the time elapsed between reaching a clinical score >3, together with having high levels of detectable viremia, and meeting endpoint criteria was seen in subjects challenged with a high dose via the conjunctival route. However, 1 animal with low viremia (6.5× 10^2^ PFU/mL of serum) at day 6 met euthanasia criteria on day 9 with no detectable viremia by plaque assay, and one animal never had viremia detected by plaque assay (Fig. 2B). qRT-PCR detected viral RNA in the serum of all animals in this group from day 6 postinfection until they met endpoint criteria (Fig. 2D). Both animals from the low dose conjunctival challenge group had low viremia by plaque assay on day 15 postchallenge and high viremia by plaque assay with RNA detectable by qRT-PCR at the time of euthanasia on day 18 postchallenge.

**FIG 3:**
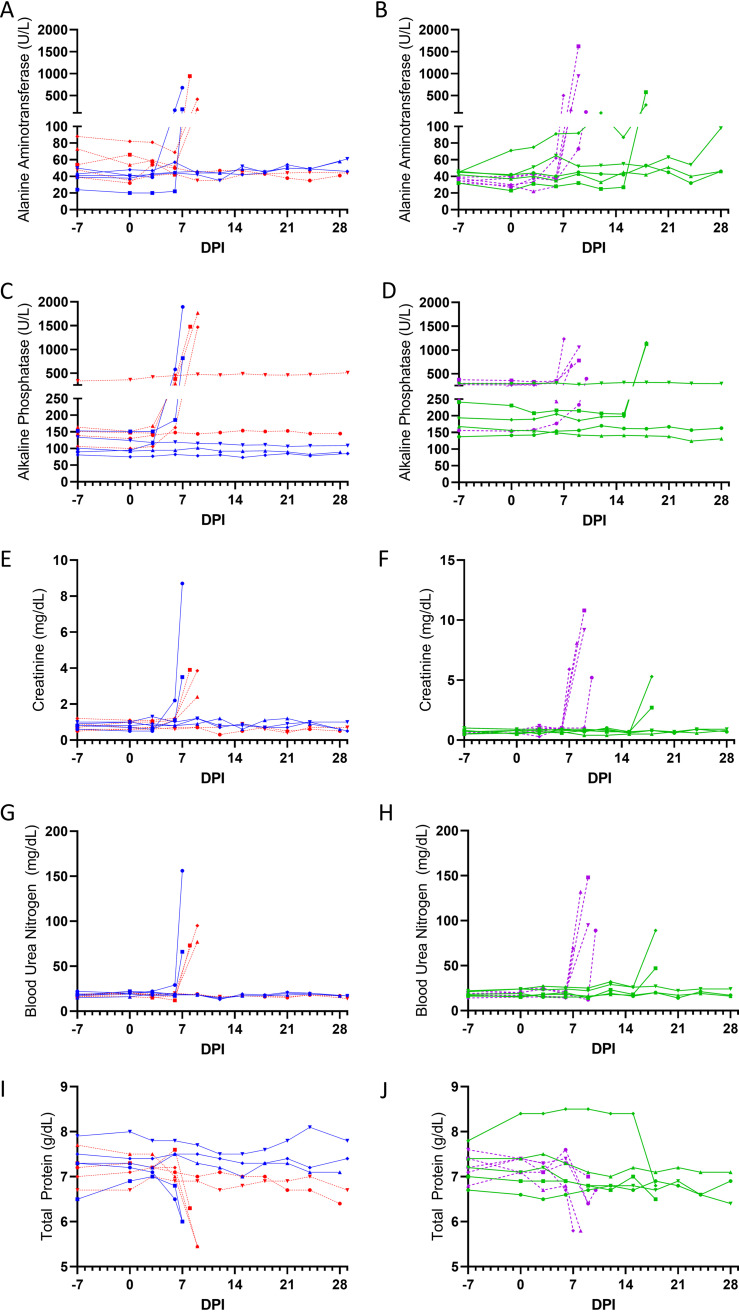
Serum was collected at an interval of 3 days following challenge and prior to euthanasia and analyzed with Comprehensive Diagnostic Profile panel on a VetScan VS2. Alanine aminotransferase (A and B), alkaline phosphatase (C and D), creatinine (E and F), blood urea nitrogen (G and H), and total protein (I and J) from NHPs challenged via the sublingual mucosa (A, C, E, G, and I) or via the conjunctiva (B, D, F, H, and J) with 1 × 10^2^ PFU (blue and green with solid connecting lines) or 1 × 10^4^ PFU (red and purple with dashed connecting lines) of EBOV. Symbols (circles, squares, triangles, diamonds, and inverted triangles) can be attributed to individual animals with the key in Fig. 7.

Clinical chemistry values support a pattern of EVD-like hepatic dysfunction apparent as elevated alanine aminotransferase (Fig. 3A and B) and alkaline phosphatase (Fig. 3C and D) which were particularly high in terminal samples. There were also signs of renal disease, including elevated creatinine (Fig. 3E and F) and blood urea nitrogen (Fig. 3G and H) in terminal samples. There were also signs of vascular leakage evident as a decrease in total protein in terminal samples (Fig. 3I and J). Similar trends of clinical chemistry were observed regardless of the route of infection (Fig. 3 and Fig. S1).

**FIG 4:**
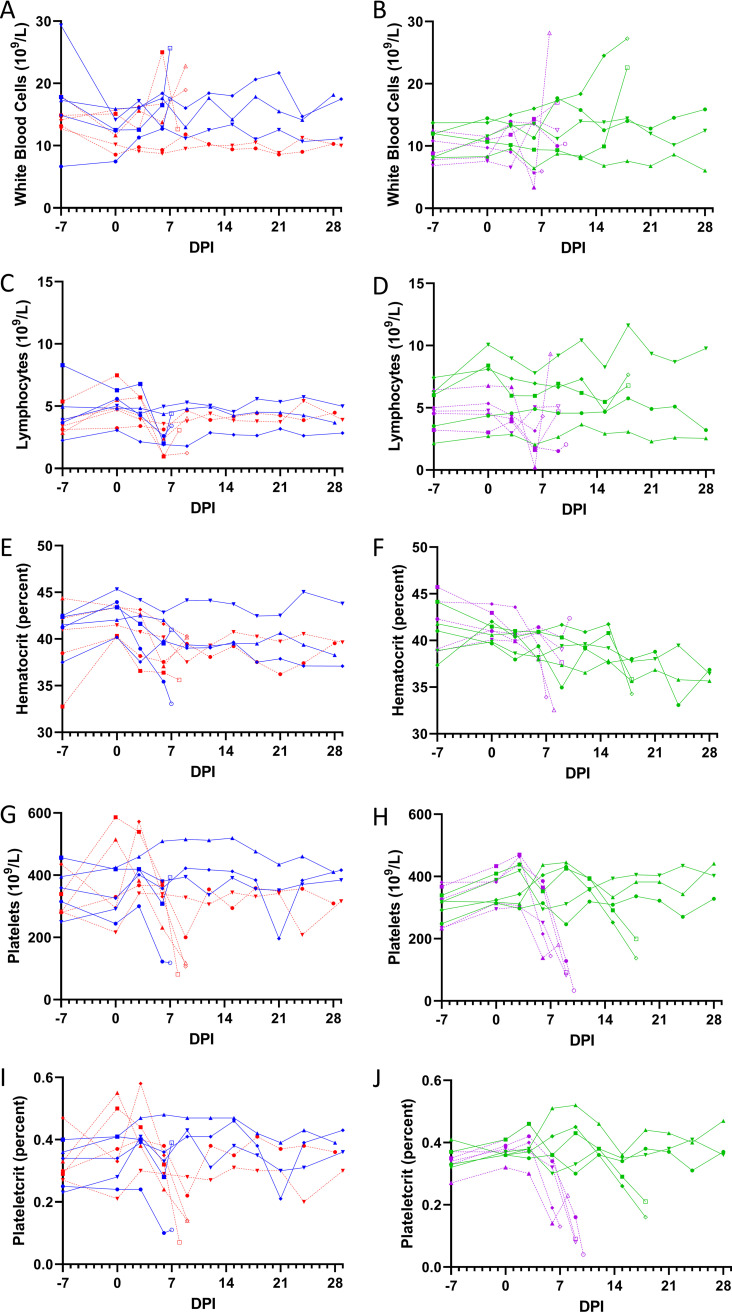
Blood was collected in EDTA tubes at an interval of 3 days following challenge and prior to euthanasia and analyzed with a VetScan HM5. White blood cells (A and B), lymphocytes (C and D), hematocrit (E and F), platelets (G and H) and plateletcrit (I and J) from NHPs challenged via the sublingual mucosa (A, C, E, G, and I) or via the conjunctiva (B, D, F, H, and J) with 1 × 10^2^ PFU (blue and green with solid connecting lines) or 1 × 10^4^ PFU (red and purple with dashed connecting lines) of EBOV. Open symbols represent terminal measurements. Symbols (circles, squares, triangles, diamonds, and inverted triangles) can be attributed to individual animals with the key in Fig. 7.

**FIG 5:**
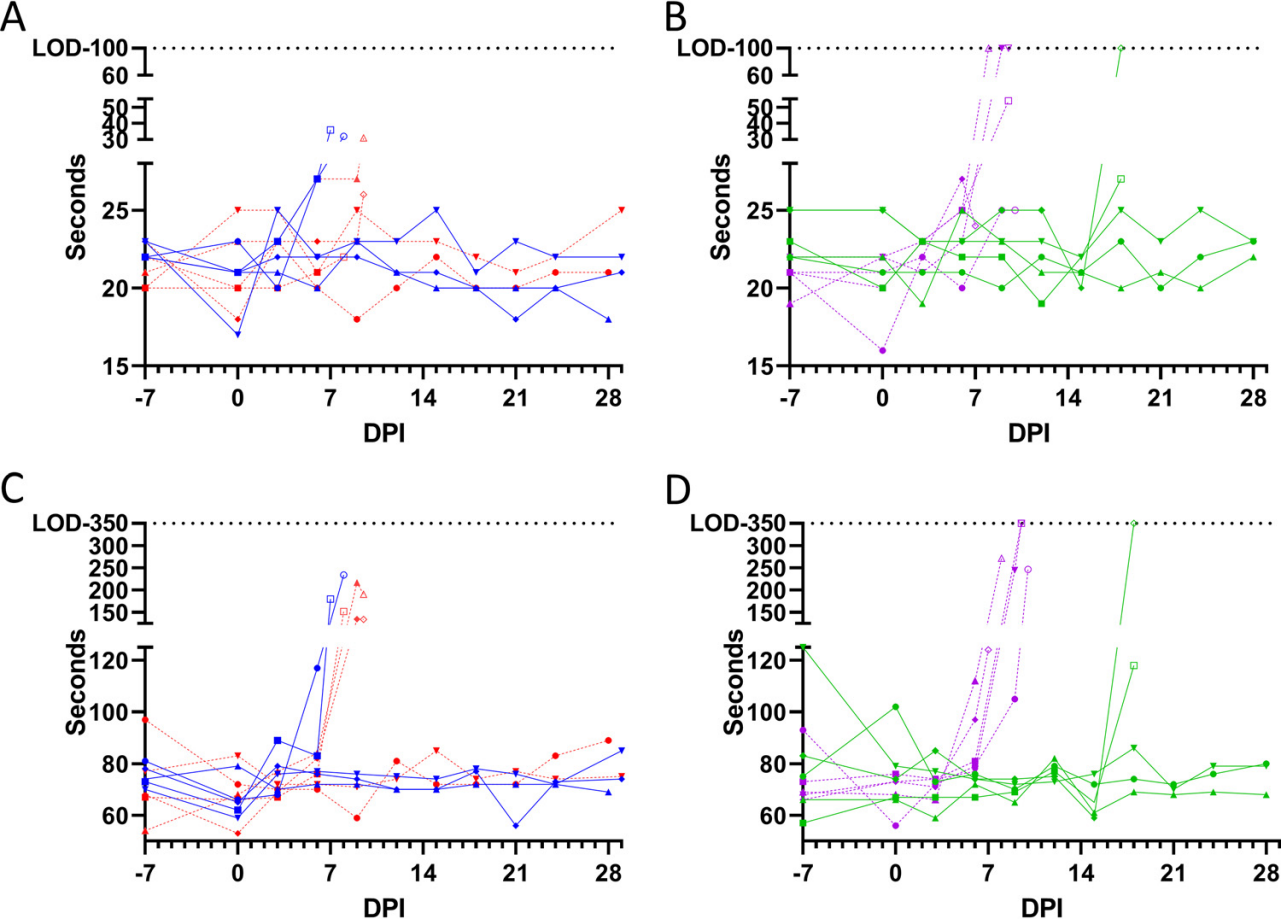
Blood was collected in sodium citrate tubes at an interval of 3 days following challenge and prior to euthanasia and analyzed with an IDEXX Coag Dx analyzer. PT (A and B) and aPTT (C and D) from NHPs challenged via the sublingual mucosa (A, C, E, G, and I) or via the conjunctiva (B, D, F, H, and J) with 1 × 10^2^ PFU (blue and green with solid connecting lines) or 1 × 10^4^ PFU (red and purple with dashed connecting lines) of EBOV. Open symbols represent terminal measurements. Symbols (circles, squares, triangles, diamonds, and inverted triangles) can be attributed to individual animals with the key in Fig. 7.

There was a slight trend toward higher white blood cell counts in NHPs following EBOV challenge (Fig. 4A and B), particularly in animals that succumbed to infection. Lymphopenia was apparent in some NHPs meeting early endpoint criteria (Fig. 4C and D). Signs disseminated intravascular coagulation (DIC), a characteristic of late-stage EVD, were evident in animals reaching endpoint criteria as low hematocrit (Fig. 4E and F), red blood cells, hemoglobin (Fig. S2), platelets (Fig. 4G and H) and plateletcrit (Fig. 4I and J). Prolonged PT and aPPT in subjects meeting endpoint criteria are congruent with EVD-like DIC (Fig. 5).

**FIG 6:**
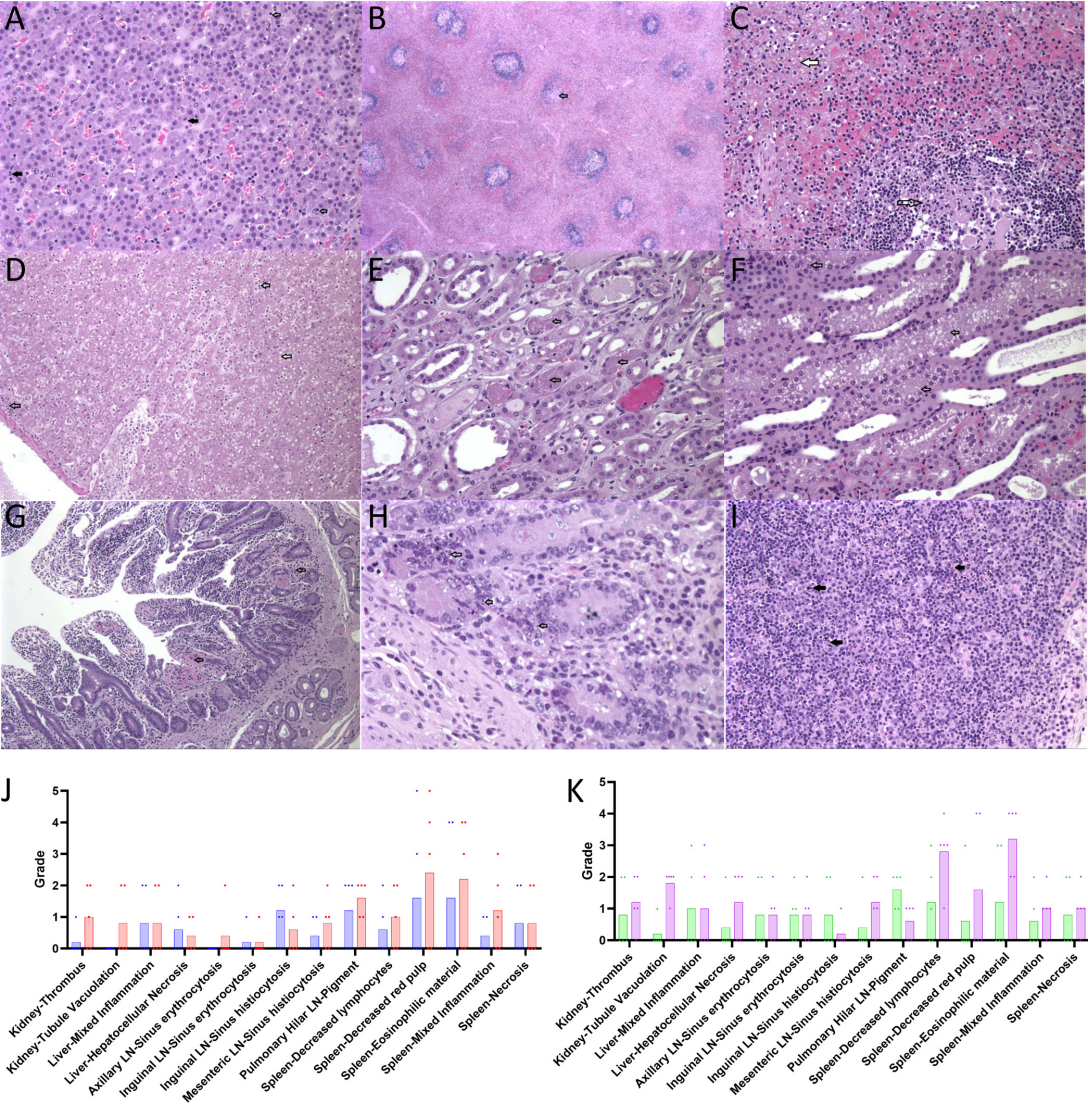
(A) 1 × 10^2^ PFU Conjunctival (Animal C96758) 20× Adrenal Gland. There are neutrophils (black arrows) and mononuclear cells (open arrows) scattered throughout the adrenal cortex. (B) 1 × 10^4^ PFU Conjunctival (Animal C96755) 2× Spleen. There are decreased lymphocytes in lymphoid nodules. (C) 1 × 10^4^ PFU Conjunctival (Animal C96755) 20× Spleen. There is lymphoid necrosis (open arrow) within lymphoid nodules and decreased red pulp (white arrow). (D) 1 × 10^4^ PFU Conjunctival (Animal C96755) 10× Liver. There is mild mononuclear inflammation (open arrow) and marked hepatocellular vacuolation (white arrow) throughout the liver. (E) 1 × 10^4^ PFU Conjunctival (Animal C99227) 20× Kidney. There are vascular thrombi (arrows) throughout vessels in the medulla. (F) 1 × 10^4^ PFU Conjunctival (Animal C99227) 20× Kidney. Renal cortical tubules contain cytoplasmic vacuoles (arrows). (G) 1 × 10^4^ PFU Conjunctival (Animal C99227) 10× Duodenum. There is multifocal mucosal hemorrhage (arrows) within the duodenum. (H) 1 × 10^4^ PFU Conjunctival (Animal C96755) 40× Jejunum. There is multifocal apoptosis (arrows) of crypt epithelium throughout the jejunum. (I) 1 × 10^4^ PFU Conjunctival (Animal C96755) 20× Mandibular lymph node. There is multifocal lymphoid necrosis (arrows) throughout the mandibular lymph node. J and K highlight all (EVD-related and non-related) graded findings with an overall mean grade greater than 0.5 of orally challenged (J) and conjunctively challenged (K) NHPs who received 1 × 10^2^ PFU (blue circles in J and green triangles in K) or 1 × 10^4^ PFU (red squares in J and purple inverted triangles in K) with the mean grade of the group represented by the bar.

Histological findings typical of EVD were observed in NHPs meeting endpoint criteria (Fig. 6). Detailed histological findings are presented in the supplementary text.

## DISCUSSION

The Kikwit strain of EBOV used here, isolated from an outbreak in 1995 (32), is the most commonly used EBOV strain in NHP challenge studies (29, 30). This is the first study to examine the Kikwit strain in an NHP oral or conjunctival model as previous studies have used the Mayinga or Makona strains. Additionally, the dosing here bridges gaps in the previous studies and can be used to inform further investigation of mucosal transmitted EBOV. For IM challenge, as little as 0.5 PFU of EBOV in cynomolgus macaques resulted in uniformly lethal disease (in 10/10 NHPs) between 7- and 10-days postchallenge (33). NHP IM models of EVD typically have a 100% CFR in untreated subjects following about a 6-day prodrome; this closely models EVD cases transmitted by IM inoculation which also have a 100% CFR and a 6-day prodrome (19, 33). In the case of contact transmission, EVD tends to have a longer prodrome of 6 to 12 days and CFRs of 35 to 86% (33). The 40%, 60%, and 40% CFR observed in the oral low dose, oral high dose, and conjunctival low dose groups, respectively, are more like the CFR typically observed from contact transmission of EVD.

Two previous studies report NHP models of oral EBOV transmission (26, 27). In the first, the Mayinga strain of EBOV was used to challenge 4 rhesus macaques. The challenge dose was administered by saturating a swab with 1 mL of virus inoculum containing 1.58x10^5^ PFU of virus and gently swabbing the oropharynx. In this study, two animals succumbed to infection at 7 to 8 days postchallenge, one animal never showed any signs of infection, and no virus or antiviral antibodies were detected in any samples, and one animal underwent intensive supportive therapy from days 9 to 12 including platelet transfusion, hydration support, and antibiotic therapy, but died at 12 day (26). In the second study, two cynomolgus macaques received 10 PFU of the Makona strain of EBOV by droplet administration to the oropharynx and survived to the end of the study with one developing low anti-EBOV-GP antibody titers, while a single NHP that received a 1 × 10^2^ dose died at 8 days postinfection (27). In the case of the first study, despite the mode of administration making it difficult to discern the actual challenge dose received, it is likely the high dose contributed to the 75% observed CFR. Alternatively, there may be a random aspect to generating aerosols during the oral inoculation and aerosol doses between 1 and 10 PFU of EBOV have a dose dependent lethal outcome (with doses greater than 10 PFU being uniformly lethal) (34–36). This could also potentially explain the reduced prodrome in the oral challenge NHP model of EBOV infection. In this study, the oral dose was administered sublingually to reduce the generation of aerosols in the oropharynx during administration. The data presented here highlight that the NHP model more faithfully reproduces mucosal transmission of EVD in humans than the ferret model which demonstrated 100% CFR for oral doses of 1 PFU of EBOV or greater and 0% CFR for ocular challenge with 100 PFU of EBOV (25).

For an arenavirus viral hemorrhagic fever rhesus macaque model, intragastric administration of virus with 4 or 5 log doses higher than the uniformly lethal intravenous (IV) dose resulted in mortality in 1 of 4 NHP, but only protected from subsequent disease following IV dosing in 1 of 3 NHPs (37). In this study, antibody titers were not detectable in the 2 NHPs that later succumbed to subsequent IV challenge (37). In this study, sublingual EBOV challenge may have led to a greater effective mucosal dose compared to previous studies where virus inoculum was applied to the oropharynx. Application to the oropharynx complicates the model as it introduces the dynamics of infection along the esophageal mucosa, the potential for droplet inhalation during administration which could unwittingly create an artificial aerosol type exposure, the potential for the mechanical disruption of transmission by the (potentially combined) action of thicker mucus layers and eventual destruction of virions by gastric acid in the stomach. There were not significant differences observed in the mean time to death (MDT) depending on dose in this study and the limited data from these three studies together does not support a relationship between dose and mean time to death. However, the limited sample size and varied study designs prohibit drawing definitive conclusions about this relationship.

Three previous studies report NHP models of conjunctival EBOV infection (26–28). In the first study, a dose of 1.58x10^5^ PFU of the Mayinga strain of EBOV was applied in 1 mL of volume dropwise to the eyes of rhesus macaques. While it was noted that most of the volume did not remain on the eye and was blotted away with gauze during the procedure, 4 of 4 NHPs succumbed to lethal infection at days 7 to 8 postinfection (26). The observed disease was highly similar to the high dose conjunctival challenge reported here. In the second and third studies, the Makona strain of EBOV was used with no mortality in 2 NHPs receiving 10 PFU, mortality in 3 of 14 animals receiving 1-5x10^2^ PFU at days 8 and 11, and uniform mortality in 6 NHPs receiving 1 × 10^4^ PFU between days 9 and 17 (27, 28). While there is a slight trend in MTD based on target inoculum titer across these previous studies and the data presented here (R^2^ = 0.4227; R^2^ = 0.3579 when adjusted for actual dose where available), a dose response is insufficient to explain the varied MTD. Interestingly, the overall range of 8–18 days for MTD in conjunctival experimental EBOV infection in NHPs is closer to the reported MTD range of 12 to 24 days of time to outcome for lethal cases of contact transmitted EVD compared to experimental IM infection in NHPs (33). It is likely that the MTD in conjunctival models of EBOV infection of NHPs are more representative of typical EVD disease process.

Interestingly, all survivors from both doses of oral EBOV challenge here had detectable circulating RNA and one animal had detectable viremia by plaque assay at the study endpoint. While the detection of RNA following EVD recovery is not uncommon (38) this would seem to indicate that all of the oral challenge NHPs were successfully infected, even if they did not develop clinically detectable disease. Persistent infection has been described in the eyes and testes of EVD survivors and has been associated with the sexual transmission of EVD (39–43). In previous studies, only 3 out of 12 NHPs surviving EBOV conjunctival developed antibody titers to EBOV-GP, and 5 of 10 tested had titers to whole inactivated EBOV (27, 28). It is possible that the EVD-like MTD in the conjunctival challenge model could be due to initial infection of the immune-privileged retinal pigment epithelial as a precursor to development of viremia.

Mucosal challenge of NHPs with EBOV results in EVD-like disease with some pathogenesis characteristics that more closely model EVD than IM challenge models. While this study is limited by the group sizes, it provides important insight into modeling transmission of human EVD using NHPs. These mucosal challenge models could be used for future studies of EVD transmission and persistence.

## MATERIALS AND METHODS

**FIG 7:**
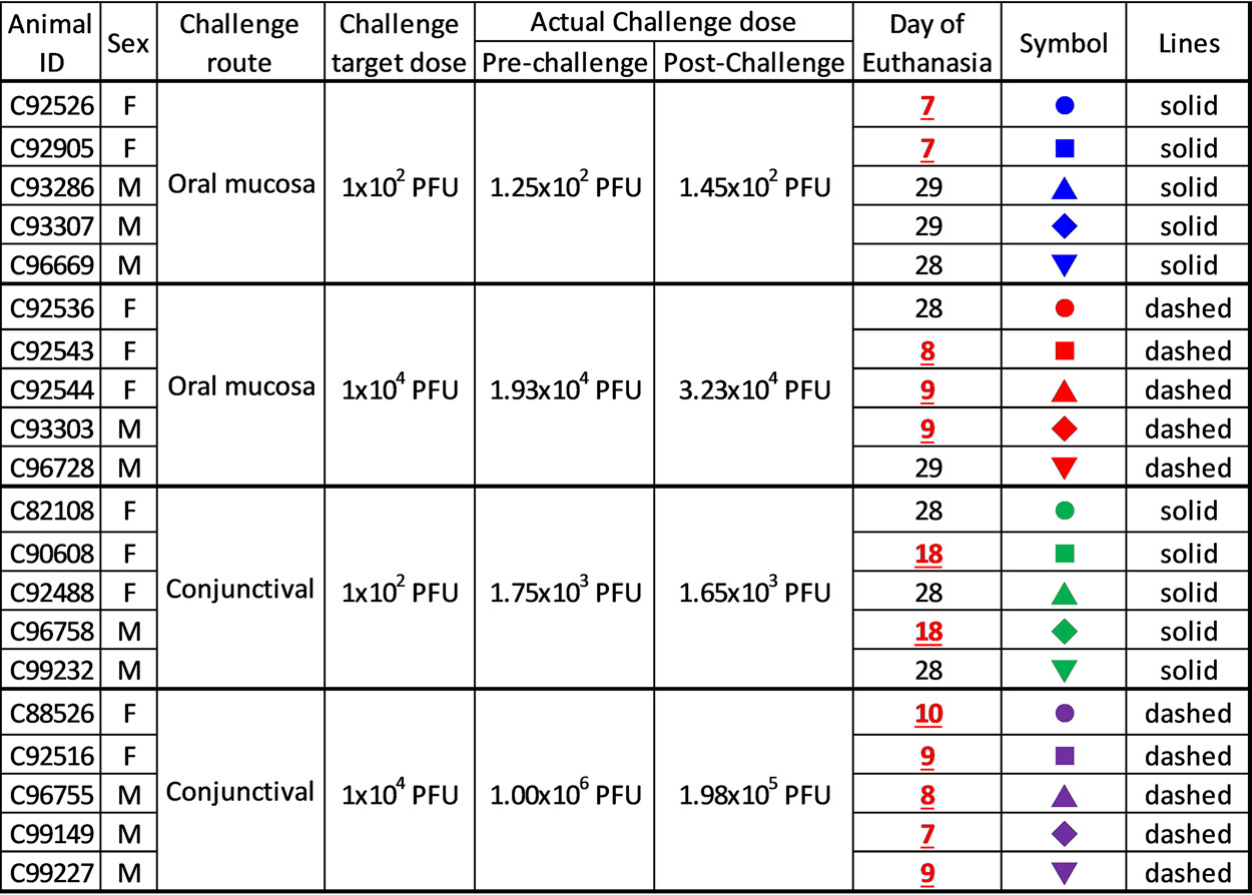
Grouping, sex, challenge parameters, and survival of NHPs.

A total of 20 NHPs were randomized into four groups of *n* = 5 and were challenged with a target dose of either 1 × 10^2^ (low dose) or 1 × 10^4^ (high dose) PFU of EBOV Kikwit strain either via pipetting under the tongue or as an evenly split dose onto the conjunctiva of each eye as indicated (Fig. 7). Following challenge, clinical observations were made a minimum of twice NHPs meeting endpoint criteria were humanely euthanized. Any surviving animals were euthanized on day 28 or day 29. Necropsy was conducted and tissues were collected in 10% neutral buffered formalin for fixation.

Fixed tissues were processed to hematoxylin and eosin-stained slides and examined by a board-certified pathologist who was blinded to groups and was unaware of the treatment group status of individual animals during the microscopic evaluation. Findings were graded from one to five, depending upon severity or graded zero in the case of nothing abnormal discovered.

Clinical chemistry analyses were conducted on harvested serum. Hematology was performed on EDTA blood. Prothrombin time (PT) and activated partial thromboplastin time (aPTT) were measured with an IDEXX Coag Dx analyzer (IDEXX Laboratories, Westbrook, ME, USA). Plaque assays were conducted on serum as described previously (31). RNA extracted from serum in TRIzol LS reagent was isolated using the Zymo Research Direct-zol RNA MiniPrep kit (Zymo Research, Irvine, CA, USA). RNA samples were analyzed via qRT-PCR targeting the EBOV glycoprotein (GP) gene.

Given the small sample size per group, all analyses were descriptive and explorative. Kaplan-Meier method was used to estimate the survival function, the probability that the animal survives over time after challenge. Figures were produced with GraphPad Prism version 9 (GraphPad Software, San Diego, CA, USA). All researchers involved in data collection were blinded to group assignment during the *in vivo* phase of the study.

The animal research protocols were approved by the UTMB IACUC and were performed in an Association for Assessment and Accreditation of Laboratory Animal Care accredited laboratory in strict accordance with the recommendations in the Guide for Care and Use of Laboratory Animals, Eighth Edition (National Academy Press, Washington, DC, USA, 2011).
