# Supplementary material for: Characterization of Ebola Virus Mucosal Challenge Routes in Cynomolgus Macaques
Source: J Virol. 2023 Mar 28;97(5):e01888-22. doi: 10.1128/jvi.01888-22 (PMC10231233; doi:10.1128/jvi.01888-22)
Supplement: Supplemental file 1 — Supplemental text, Fig. S1 and S2, and clinical scoring sheet. Download jvi.01888-22-s0001.pdf, PDF file, 0.5 MB [file jvi.01888-22-s0001.pdf]

Online Only Supplemental Text – Description of Histological Findings

In NHPs challenged with $1x10^2$ PFU of EBOV via the oral route that met early euthanasia criteria, microscopic findings included mild neutrophilic cortical inflammation of the adrenal gland; minimal to mild decreased lymphocytes, moderate to severe decreased red pulp, marked eosinophilic material, minimal mixed inflammation and mild necrosis in the spleen; moderate mononuclear inflammation in the lung; minimal to mild mixed inflammation and hepatocellular necrosis of the liver; minimal to mild hemoglobin cast and minimal thrombus in the kidney; minimal to mild gastric mucosal hemorrhage; mild duodenal mucosal hemorrhage; minimal lymphoid necrosis of the mesenteric lymph node; mild decreased lymphocytes and minimal to mild lymphoid necrosis of the tonsil; and minimal to mild lymphoid necrosis of pulmonary hilar, axillary, mandibular, mesenteric, and inguinal lymph nodes. For NHPs challenged with $1x10^4$ PFU of EBOV meeting early euthanasia criteria microscopic findings included mild mixed cortical inflammation of the adrenal gland; minimal to mild mixed or neutrophilic inflammation of the adrenal cortex; minimal to mild decreased lymphocytes, marked to severe decreased red pulp, marked eosinophilic material, minimal to mild mixed inflammation and mild necrosis of the spleen; mild mixed inflammation, mild mononuclear inflammation, and minimal hepatocellular necrosis of the liver; minimal thrombus of the lung; minimal hemoglobin cast, minimal to mild thrombus and mild vacuolation of the kidney; minimal gastric mucosal hemorrhage; mild submucosal hemorrhage and apoptosis in the cecum; minimal apoptosis of the ileum; mild neutrophilic inflammation and mild lymphoid necrosis of the mesenteric lymph node; minimal to mild lymphoid necrosis of the inguinal lymph node; minimal lymphoid necrosis of the pulmonary hilar lymph node; minimal lymphoid necrosis of the axillary lymph node; mild lymphoid necrosis of the mandibular lymph node; minimal decreased lymphocytes of the tonsil; and mild mucosal and submucosal hemorrhage of the duodenum. A summary of microscopic histology findings from these groups is included as Figure 6J.

In the male NHPs challenged with $1x10^2$ of EBOV via the conjunctival route that met early euthanasia criteria microscopic findings included mild mixed (neutrophils and mononuclear cells)

inflammation of the cortex (Figure 6A) and mild mononuclear inflammation of the medulla of the adrenal gland; mild decreased lymphocytes, moderate decreased red pulp, moderate eosinophilic material and mild mixed inflammation of the spleen; mild mixed inflammation of the liver; moderate tubule necrosis and mild thrombus of the kidney; minimal gastric mucosal hemorrhage; mild hemorrhage of the testis; minimal lymphoid necrosis of the pulmonary hilar, axillary, and mandibular lymph nodes. Moderate mononuclear inflammation of the heart was also observed in the early euthanized male in this group. It is unclear if this finding is attributed to EBOV exposure as idiopathic inflammatory cell infiltrations and focal myocarditis can be a common spontaneous finding in the heart of nonhuman primates used as controls in preclinical safety studies [1, 2]. In the female challenged with $1x10^2$ of EBOV via the conjunctival route that met early euthanasia criteria microscopic findings included minimal cortical degeneration, mild neutrophilic cortical inflammation and minimal necrosis of the adrenal gland; moderate decreased lymphocytes, moderate eosinophilic material, minimal mixed inflammation and mild necrosis of the spleen; moderate mixed inflammation of the liver; mild thrombus and minimal tubule vacuolation of the kidney; minimal decreased lymphocytes and lymphoid necrosis of the mesenteric lymph node; mild lymphoid necrosis of the pulmonary hilar lymph node and minimal mucosal hemorrhage of the bladder and rectum. In males challenged with $1x10^4$ PFU of EBOV via the conjunctival route microscopic findings included minimal mixed cortical inflammation of the adrenal glands; moderate decreased lymphocytes, marked decreased red pulp, mild to marked eosinophilic material and minimal necrosis of the spleen (Figure 6 B and C); mild mononuclear inflammation of the pleura of the lung; mild to moderate mixed or mononuclear inflammation, mild hepatocellular necrosis and marked hepatocellular vacuolation of the liver (Figure 6D); mild tubular necrosis, minimal to mild thrombus and minimal to mild tubule vacuolation of the kidney (Figure 6 E and F); minimal apoptosis and mild mucosal hemorrhage of the duodenum (Figure 6G); minimal apoptosis of the cecum; mild apoptosis of the jejunum (Figure 6H); minimal to mild apoptosis and minimal mucosal hemorrhage of the ileum; minimal lymphoid necrosis and thrombus of the inguinal lymph node; minimal decreased lymphocytes and lymphoid necrosis of the pulmonary hilar lymph node; mild lymphoid necrosis of

the axillary lymph node; mild lymphoid necrosis (Figure 6I) and mixed inflammation of the mandibular lymph node and mild submucosal hemorrhage of the rectum. In females challenged with $1x10^4$ PFU of EBOV via the conjunctival route, microscopic findings included minimal mixed cortical inflammation of the adrenal gland, minimal to marked decreased lymphocytes, mild to marked eosinophilic material, minimal to mild mixed or mononuclear inflammation and mild necrosis of the spleen; mild thrombus of the lung; minimal to mild inflammation and mild hepatocellular vacuolation of the liver; minimal to mild thrombus and mild tubule vacuolation of the kidney; mild apoptosis of the jejunum, colon, and ileum; minimal lymphoid necrosis of the mesenteric, inguinal, pulmonary hilar and axillary lymph node; mild decreased lymphocytes and lymphoid necrosis of the mandibular lymph node; mild thrombus of the choroid of the eye and minimal mucosal hemorrhage of the rectum. Decreased red pulp in the spleen of affected groups may be due to replacement by inflammatory cells or a difference in exsanguination between animals. The nature of the splenic eosinophilic material is unclear but may be a more prominent reticular network due to the absence of red pulp or may be fibrin; special stains may assist in elucidating the nature of the material. A summary of microscopic histology findings from these groups is included as Figure 6K.

Online Only Supplemental Text – Detailed Materials and Methods

The EBOV Kikwit strain (Ebola virus/H. sapiens-tc/COD/1995/Kikwit-9510621, accession number MH121165.1) was passaged 3 times in Vero E6 cells (BEI Recourses, NR-596) and verified by deep sequencing which did not reveal evidence of the 8U mutation. Viral stocks had a particle-to-PFU count of 53:1 determined using a ViroCyte 2100 Virus Counter (ViroCyt, LLC), tested mycoplasma free using a MycoAlert Mycoplasma Detection Kit (Lonza), had endotoxin levels <0.050 EU/ml determined with the Endosafe-PTS System (Charles River), and had documented sterility on Sabouraud Dextrose Agar, Tryptic Soy Agar with defibrinated Sheep blood, Dulbecco's Modified Eagle Medium, and Thioglycolate broth.

Specific pathogen free Chinese origin cynomolgus macaques were obtained from Covance (Alice, TX) and underwent a quarantine period and veterinary physical exam prior to transfer to individual housing at ABSL4. Certified Primate Diet (PMI, Inc.) was provided to the animals daily. Drinking water was provided ad libitum through an automatic watering system. To promote and enhance the psychological well-being of the NHPs, both food and environmental enrichment were provided to all NHPs. Prior to handling, NHPs were anesthetized with an IM injection of ketamine. Body weight, rectal temperature, and hydration status were measured at each anesthetic event.

A total of 20 NHPs were randomized into four groups of n = 5 and were challenged with a target dose of either $1x10^2$ (low dose) or $1x10^4$ (high dose) PFU of EBOV either via pipetting under the tongue or as an evenly split dose onto the conjunctiva of each eye as indicated (table 1). Viral stocks were diluted in Dulbecco's phosphate buffered saline without divalent cations to a volume of 0.5 mL for oral challenge or 0.02 mL for conjunctival challenge. Aliquots of the diluted challenge doses were collected before and after administration and were tittered by plaque assay as described below. All researchers involved in data collection were blinded to group assignment during the *in vivo* phase of the study.

Following challenge, clinical observations were made a minimum of twice daily using an internal scoring system approved by the University of Texas Medical Branch (UTMB) Institutional Animal Care and Use Committee (IACUC) that includes respiration, food consumption, excretion of urine and feces, activity, appearance, and signs of bleeding or hemorrhage. NHPs meeting endpoint criteria based on clinical scoring were humanely euthanized in accordance with the current version of the American Veterinary Medical Association guidelines for the euthanasia of animals. Any surviving animals were euthanized on day 28 or day 29.

Necropsy was conducted and tissues were collected in 10% neutral buffered formalin for fixation. Fixed tissues were processed to hematoxylin and eosin-stained slides and examined by a board-

certified pathologist who was blinded to groups and was unaware of the treatment group status of individual animals during the microscopic evaluation. Findings were graded from one to five, depending upon severity or graded zero in the case of nothing abnormal discovered.

Femoral vein peripheral blood was collected via Vacutainer® into standard collection tubes containing a clot activator (serum separator tubes), EDTA, or sodium citrate. Clinical chemistry analyses were conducted on harvested serum using the Abaxis VetScan VS2® Chemistry Analyzer with Comprehensive Diagnostic Profile rotors (Abaxis, Inc., Union City, CA, USA). Hematology was performed on EDTA blood using the Abaxis VETSCAN® HM5 Hematology Analyzer (Abaxis, Inc., Union City, CA, USA). Prothrombin time (PT) and activated partial thromboplastin time (aPTT) were measured with an IDEXX Coag Dx analyzer (IDEXX Laboratories, Westbrook, ME, USA). Aliquots of serum were frozen for viral load analysis by plaque assay or stored frozen in TRIzol LS reagent (Thermo Fisher Scientific, Waltham, VA, USA) viral load analysis by quantitative reverse-transcriptase polymerase chain reaction (qRT-PCR). Plaque assays were conducted as described previously [31]. Briefly, serum was thawed, serially diluted. For plaque assays, serially diluted serum was absorbed onto Vero E6 cells for 1 hour prior to overlay with Modified Eagles Medium containing 5% fetal bovine serum and 0.5% agarose. 10 days later, additional overlay containing 5% neutral red was added and plates were incubated for an additional 12-24 hours before plaques were counted.

RNA was extracted from serum in TRIzol LS reagent using the Zymo Research Direct-zol™ RNA MiniPrep kit (Zymo Research, Irvine, CA, USA) per manufacturer instructions. RNA samples were analyzed via qRT-PCR targeting the EBOV glycoprotein (GP) gene (Forward: 5’-TTT TCA ATC CTC AAC AAG CGT GC-3’; Reverse: 5’-CAG TCC GGT CCC AGA ATG TG-3’; Probe: 5’-6FAM-CAT GTG CCG CCC CAT CGC TGC-MGBNFQ-3’) with a Quantifast Probe PCR kit (Qiagen, Germantown, MD, USA) using a Bio-Rad CFX96™ Real-Time PCR Detection System qRT-PCR with the following reaction conditions: reverse transcription at 50°C for minutes, DNA polymerase

activation at 95°C for 5 minutes, followed by 45 cycles of 95°C for 30 seconds followed by 95°C for 1 minute. Comparison to a standard curve composed of EBOV synthetic RNA (Integrated DNA Technologies, Coralville, IA) was used to determine EBOV genetic equivalents per microliter of serum.

Given the small sample size per group, all analyses were descriptive and explorative. Kaplan-Meier method was used to estimate the survival function, the probability that the animal survives over time after challenge. Figures were produced with GraphPad Prism version 9 (GraphPad Software, San Diego, CA, USA).

The animal research protocols used in this study were performed in strict accordance with the recommendations in the Guide for Care and Use of Laboratory Animals, Eighth Edition (National Academy Press, Washington, DC, USA, 2011). The UTMB facility where these studies were conducted is accredited by the Association for Assessment and Accreditation of Laboratory Animal Care. The protocols were approved by the UTMB Institutional Animal Care and Use Committee and complied with the Animal Welfare Act, the U.S. Public Health Service Policy, and other federal statutes and regulations related to animals and experiments involving animals.

References

1. Chamanza R, Marxfeld HA, Blanco AI, Naylor SW, Bradley AE. Incidences and range of spontaneous findings in control cynomolgus monkeys (Macaca fascicularis) used in toxicity studies. Toxicol Pathol. Sage Publications Sage CA: Los Angeles, CA; **2010**; 38(4):642–657.

2. Sato J, Doi T, Kanno T, Wako Y, Tsuchitani M, Narama I. Histopathology of incidental findings in cynomolgus monkeys (macaca fascicularis) used in toxicity studies. J Toxicol Pathol. Japanese Society of Toxicologic Pathology; **2012**; 25(1):63–101.

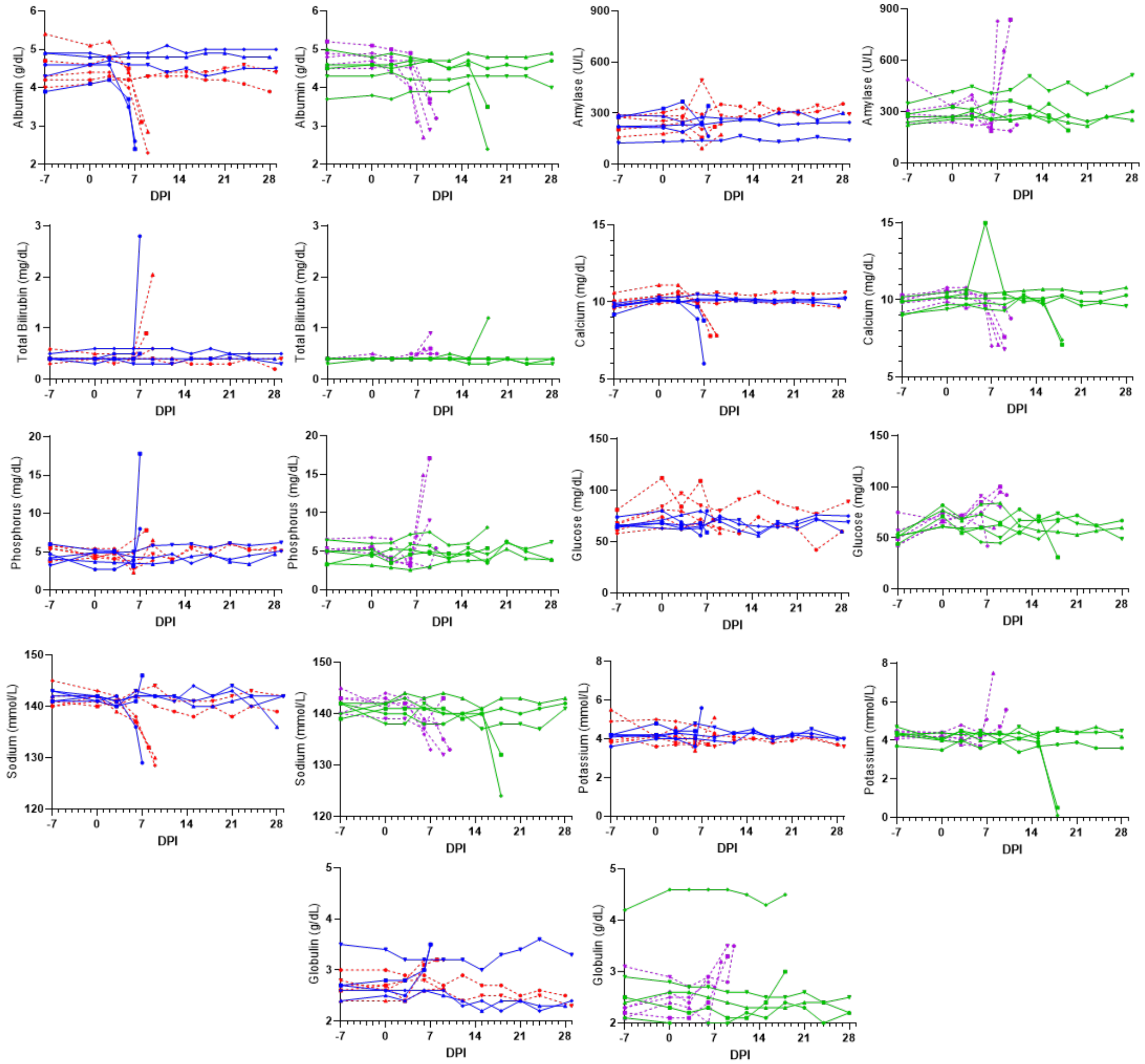

Supplemental Figure 1. Serum was collected at an interval of 3 days following challenge and prior to euthanasia from NHPs challenged via the sublingual mucosa (blue and red) or via the conjunctiva (green and purple) with 1x10^2 PFU (blue and green with solid connecting lines) or 1x10^4 PFU (red and purple with dashed connecting lines) of EBOV. Serum was analyzed with Comprehensive Diagnostic Profile panel on a VetScan VS2 for albumin, amylase, total bilirubin, calcium, phosphorus, glucose, sodium, potassium, and globulin as indicated on the y-axis.

Symbols (circles, squares, triangles, diamonds, and inverted triangles) can be attributed to individual animals with the key in table 1.

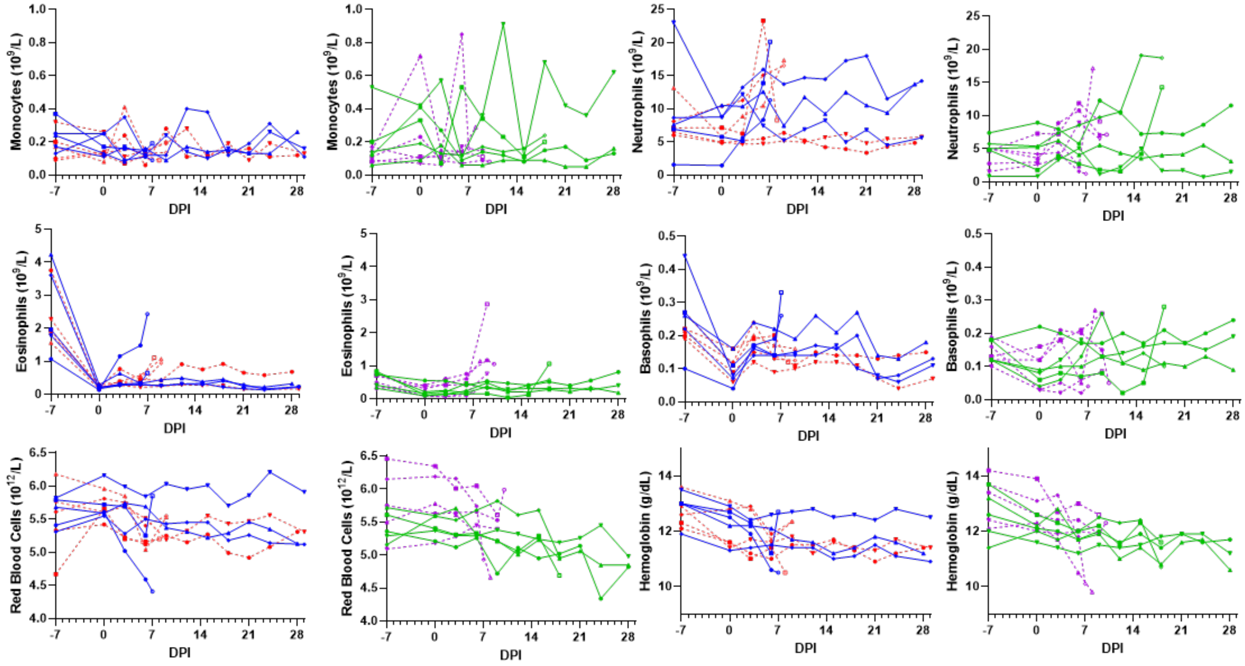

Supplemental Figure 2. Blood was collected at an interval of 3 days following challenge and prior to euthanasia from NHPs challenged via the sublingual mucosa (blue and red) or via the conjunctiva (green and purple) with 1x10^2 PFU (blue and green with solid connecting lines) or 1x10^4 PFU (red and purple with dashed connecting lines) of EBOV. Blood was analyzed with VetScan HM5 for monocytes, neutrophils, eosinophils, basophils, red blood cells, and hemoglobin as indicated in the graph titles. Symbols (circles, squares, triangles, diamonds, and inverted triangles) can be attributed to individual animals with the key in table 1.

Supplement – Clinical Scoring Sheet

Nonhuman Primate Clinical Scoring: Filoviruses

Animal Identification: ____________________ Study Day: ____________________

IACUC Protocol #: ____________________ Study Protocol #: ____________________

Parameter	Degree of Parameter	Ob 1	Ob 2	Ob 3	Ob 4
	**Observation Start Time:**				
**Parameter**	**Degree of Parameter**	**Score (Circle One)**	**Score (Circle One)**	**Score (Circle One)**	**Score (Circle One)**
Respiration	Normal	0	0	0	0
	Abdominal breathing or labored breathing	4	4	4	4
	Severe dyspnea; agonal breathing	10	10	10	10
Food Consumption Feces/Urine	Normal	0	**Value for Observation 1 (Ob 1) should be carried over for Observations 2 through 4 (Ob 2, Ob 3, and Ob 4) as applicable.**		
	No biscuits eaten = 1<br>Consecutive days (Day 2=2, Day 3=3, Day 4=4, etc.)[1]				
	No enrichment eaten = 1<br>Consecutive days (Day 2=3, Day 3=4, Day 4=5, etc.)[2]				
	No feces seen (AM check); no urine seen (AM check)	1			
	Diarrhea (liquid)	2			
Activity/ Appearance	Normal	0	0	0	0
	Hunched but active most of the time	1	1	1	1
	Hunched with head between knees; dull appearance to eyes	3	3	3	3
	Lies down; gets up when approached	4	4	4	4
	Lies down; gets up with some prodding but not when approached	10	10	10	10
Bleeding/ Hemorrhage	No signs	0	0	0	0
	Petechiation and/or ecchymosis	2	2	2	2
	Observable bleeding; controlled by clotting (not menses) **AND/OR** Petechiation and/or ecchymosis > 50%	4	4	4	4
	Uncontrolled bleeding	10	10	10	10
	**Total Score (sum of all circled values)**				
	**Respiratory Rate (breaths per minute):**				
	**Observation End Time:**				
	Observations Performed by:	(Initial/Date)	(Initial/Date)	(Initial/Date)	(Initial/Date)
	Observations Recorded by:	(Initial/Date)	(Initial/Date)	(Initial/Date)	(Initial/Date)

Score = 0-3, no intervention. Score = ≥ 4 (or ≥ 3 in any single parameter), additional monitoring of at least once in the evening 4-6 hours after the final late afternoon check. Score ≥ 10, Euthanasia. [1] consecutive days with NO biscuit consumption only. [2] consecutive days with NO enrichment consumption only

Comments (Initial/Date all comments below):

Reviewed/QC: ____________________
Initial Date
